# Supplementary material for: Reconfigurable droplet networks
Source: Nat Commun. 2024 Feb 5;15:1058. doi: 10.1038/s41467-024-45214-1 (PMC10844234; doi:10.1038/s41467-024-45214-1)
Supplement: Supplementary file 1 — Supporting Information [file 41467_2024_45214_MOESM1_ESM.pdf]

Supporting information

Reconfigurable Droplet Networks

Shuyi Sun[1], Shuailong Li[1], Weixiao Feng[1], Jiaqiu Luo[1], Thomas P. Russell*[2,3] and Shaowei Shi*[1]

1. State Key Laboratory of Chemical Resource Engineering, Beijing Advanced Innovation Center for Soft Matter Science and Engineering, Beijing University of Chemical Technology, Beijing, 100029, China

2. Department of Polymer Science and Engineering, University of Massachusetts, Amherst, Massachusetts 01003, USA

3. Materials Sciences Division, Lawrence Berkeley National Laboratory, 1 Cyclotron Road, Berkeley, California 94720, USA

Corresponding author: russell@mail.pse.umass.edu; shisw@mail.buct.edu.cn

Table of contents

Synthesis Procedures 2
Results and Discussion 5
Reference 19

Synthesis Procedures

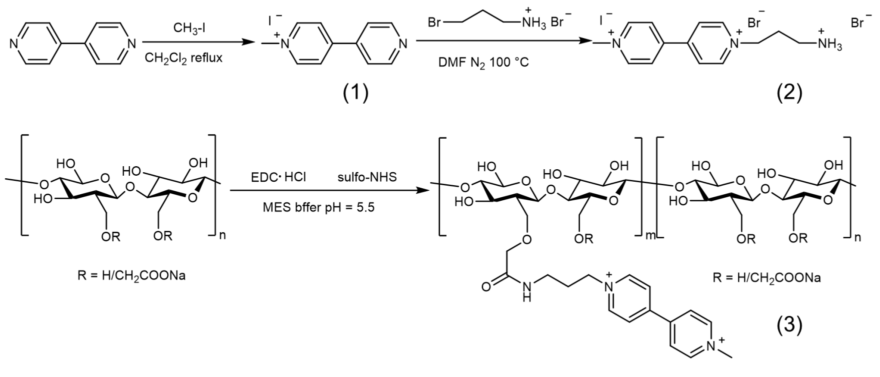

Supplementary Scheme 1. Synthesis route of CMC-MV^{2+}

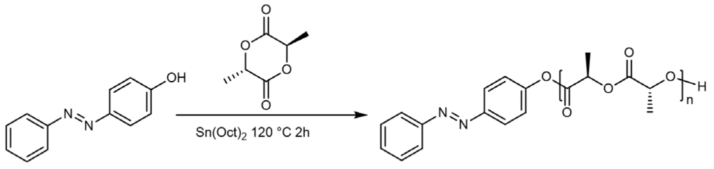

Supplementary Scheme 2. Synthesis route of Azo-PLLA

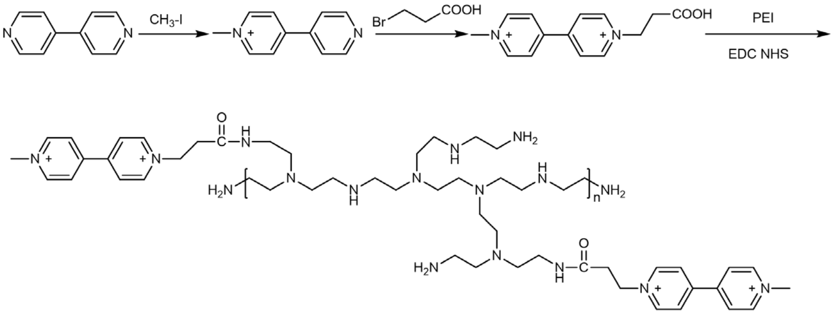

Supplementary Scheme 3. Synthesis route of PEI-MV^{2+}

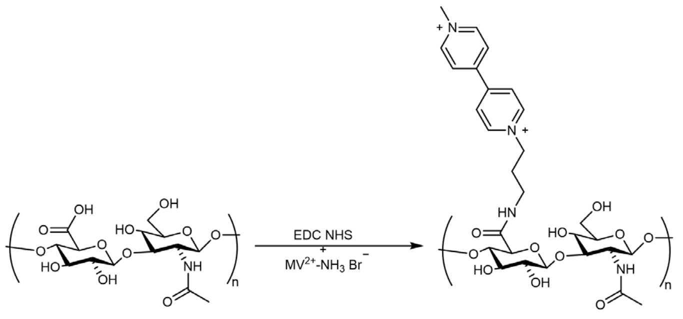

Supplementary Scheme 4. Synthesis route of HA-MV^{2+}

Synthesis of 1-Methyl-4,4’ -bipyridinium Iodide (MV^+) (1). MV^+ were synthesized according to the literature. [1] 4,4’ -Bipyridinium (2 g, 12.8 mmol) was dissolved in a round-bottom flask containing 30 mL of dichloromethane. Methyl iodide (860 μL, 13.6 mmol) in 10 mL dichloromethane was added drop-wise to the stirred flask, the mixture was heated to reflux for 6 h and left to cool with stirring. The light-yellow precipitate was filtrated and recrystallized in methanol (1.60 g, 42%). ^{1}H NMR (400 MHz, D_2O, δ): 8.9 (d, 2H), 8.7 (m, 2H), 8.4 (d, 2H), 7.9 (m,2H), 4.4 (s, 3H).

Synthesis of 1-(3-ammoniopropyl)-1'-methyl-[4,4'-bipyridine]-1,1'-diium (MV^{2+}-NH_2·HBr) (2). [2] Product (1) (0.650 g, 2.18 mmol) and 3-bromo-1-propanamine hydrobromide (1.43 g, 6.55 mmol) were mixed in 20 mL DMF. Next, the mixture was stirred at 100 °C for 20 h under N_2 atmosphere. After being cooled to room temperature, the mixture was filtered and the precipitate was washed with DMF to give a yellow solid, yielding 49%. The raw product was used in the next reaction (synthesis of CMC-MV^{2+}) without further purification. ^{1}H NMR (400 MHz, DMSO-d_6, δ): 9.5 (d, 2H), 9.3 (m, 2H), 8.9 (m, 4H), 8.1 (m, 3H), 4.8 (m, 2H), 4.5 (s, 3H), 3.3-3.2 (m, 2H), 2.3-2.2 (m,2H).

Synthesis of methyl viologen functional carboxymethylcellulose sodium salt (CMC-MV^{2+}). [2] 0.20 g of carboxymethylcellulose sodium salt (CMC, with the viscosity coefficient of 1200 cps, degree of substitution: 1.2) was dispersed in 15 mL 0.2 M MES (2-morpholinoethanesulfonic acid) buffer (pH = 5.5). The mixture was stirred at room temperature for 48 h to ensure that CMC was completely dissolved. 80

mg (0.41 mmol) of 1-ethyl-3-(3-dimethylaminopropyl) carbodiimide hydrochloride (EDC·HCl) was dissolved in 1 mL 0.2 M MES buffer. 100 mg (0.46 mmol) of N-hydroxysulfosuccinimide sodium salt (sulfo-NHS) was dissolved in 1 mL 0.2 M MES buffer and 40 mg of MV-NH_2·HBr (row product, ~ 0.11 mmol) was dissolved in 1 mL 0.2 M MES buffer. The solution of EDC·HCl and then the solution of sulfo-NHS was added dropwise into the solution of CMC rapidly with continuous stirring at room temperature. After stirring for 2 min, the solution of MV^{2+}-NH_2·HBr was added dropwise into the mixture with continuous stirring at room temperature. The mixture was stirred at room temperature for 4 h. Then the mixture was dialyzed by 1 L deionized water for 72 h (1000 Da). After dialysis, the solution was lyophilized for 48 h to give a light-yellow foam-like solid. 170 mg of product was collected.

According to elemental analysis as well as the integral of ^{1}H NMR spectrum (Supplementary Figure 3), the amount of viologen moieties in CMC-MV^{2+} was determined to be 0.62 mmol/g.

Elemental analysis: C: 41.73%, H: 5.43%, N: 2.60%

Synthesis of azobenzene functional Poly-L-lactic acid (Azo-PLLA). Azobenzene functional poly L-lactic acid (Azo-PLLA) was synthesized according to the reported method. [3] 1.0 g (7.0 mmol) L-lactic acid (LLA) and 138 mg (0.7 mmol) 4-Phenylazophenol were added into a 10 mL Schlenk flask. Three freeze-pump-thaw cycles were performed to remove the oxygen. The flask was immersed into an oil bath at 120 °C with vigorous magnetic stirring for 3.0 min. Then 2.5 μL tin 2-ethylhexanoate catalyst in dry 50 μL DMF was added to the mixture via a microsyringe. After reacting for 2.0 h at 120 °C, the crude product was dissolved in DCM, and the solution was precipitated into cold methanol. The product was washed by three times. The final product was dried in 60 °C vacuum, yielding a white solid (600 mg, 60%). FT-IR (KBr, cm^{-1}): 757 ($V_{C\text{-}H}$ in Azobenzene), 1759 ($V_{C=O}$ in PLLA), 2948 ($V_{C\text{-}H}$ in PLLA), 2997 ($V_{C\text{-}H}$ in PLLA). ^{1}H NMR spectrum (400 MHz, $CDCl_3$, δ): 7.89-7.76 (m, 4H), 7.58-7.44 (t, 2H), 7.23-7.18 (d, 1H). M_n = 5200, M_w = 5800, PDI = 1.1. NT-PLLA and PY-PLLA were synthesized in the same way of Azo-PLLA.

Synthesis of 1-(2-carboxyethyl)-1'-methyl-[4,4'-bipyridine]-1,1'-diium (MV^{2+}-COOH). [4] MV^+ (product (1)) (500 mg, 1.68 mmol) was dissolved in 50 mL acetonitrile, treated with excess 3-Bromopropionic acid (2.57 g, 16.8 mmol) and heated to reflux for

24 h. A yellow precipitate was collected by suction filtration, washed with acetonitrile, then the product was recrystallized from ethanol and dried under a vacuum overnight yielding a light yellow solid (252 mg, 33%). ^{1}H NMR spectrum (400 MHz, DMSO-d$_6$, δ): 12.8 (s, 1H), 9.3 (m, 4H), 8.8 (m, 4H), 4.9 (t, 2H), 4.5 (s, 3H), 3.2(t, 2H).

Synthesis of methyl viologen functional polyethyleneimine (PEI-MV^{2+}). [5] MV^{2+}-COOH (45 mg, 1 mmol) was first activated by EDC·HCl (100 mg, 0.51 mmol) and sulfo-NHS (30 mg, 0.14 mmol) in 2 mL of MES buffer (pH = 5.5) in an ice bath for 0.5 h. PEI (120 mg, 1 mmol of primary amine) dissolved in 2 mL MES buffer was then added. The reaction mixture was stirred at room temperature for 12 h and then purified by dialyzing against DI water for 3 days (MW cutoff 3500). The resulting solution was lyophilized for 48 h, and the light-yellow product was finally obtained (100 mg).

Synthesis of methyl viologen functional hyaluronic acid (HA-MV^{2+}). The synthesis method is similar with that of CMC-MV^{2+}.

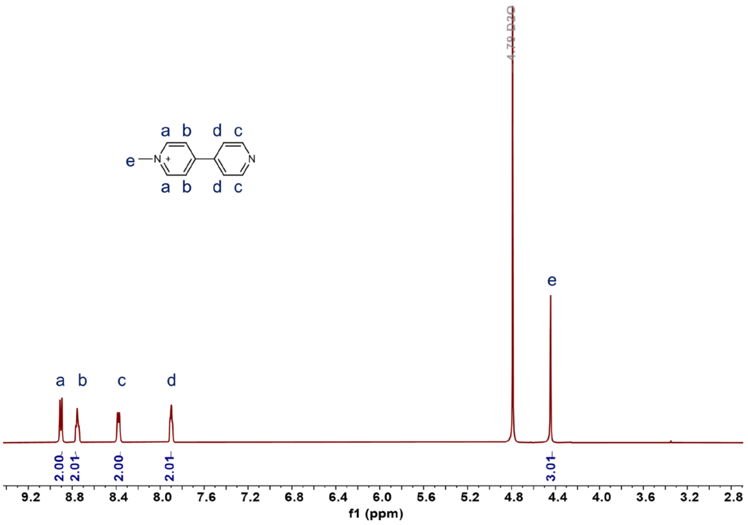

Supplementary Figure 1. ^{1}H NMR spectrum of MV$^+$ in D$_2$O.

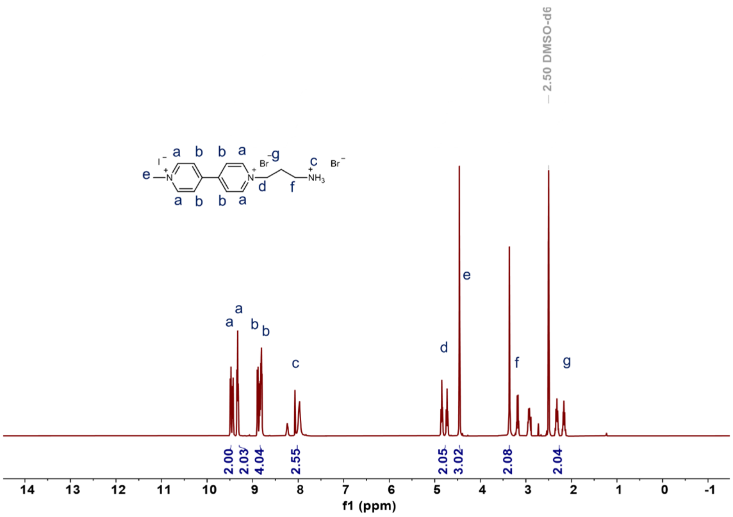

Supplementary Figure 2. ^{1}H NMR spectrum of MV^{2+}-NH_2·HBr in DMSO-d_6.

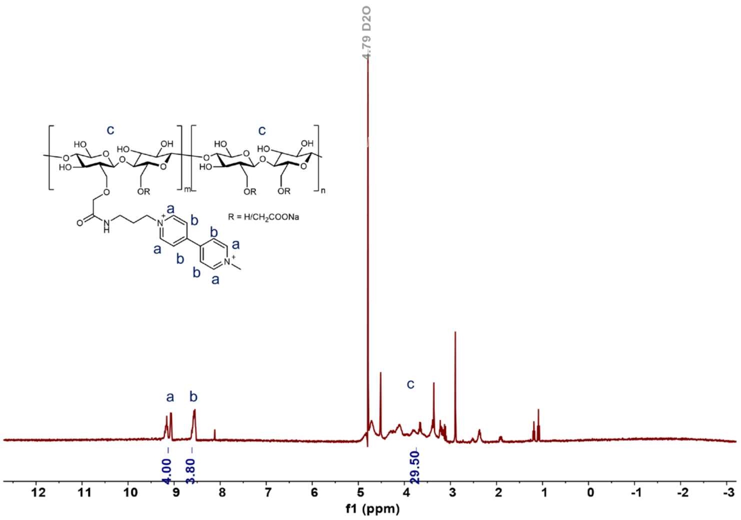

Supplementary Figure 3. ^{1}H NMR spectrum of CMC-MV^{2+} in D_2O.

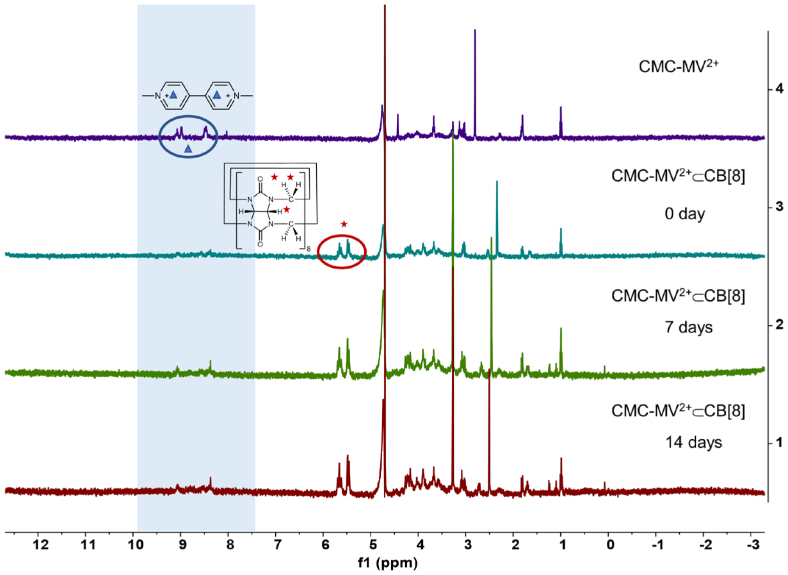

Supplementary Figure 4. ^{1}H spectra (400 MHz D_2O) of CMC-MV^{2+} and CMC-MV^{2+}⊂CB[8] binary complex.

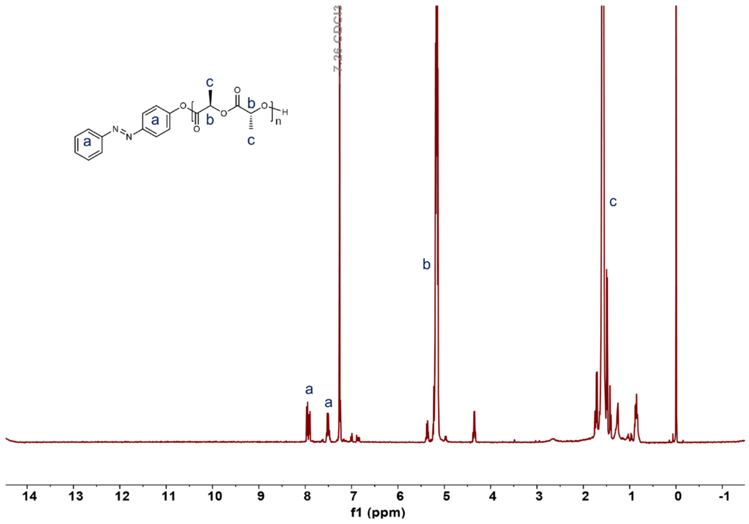

Supplementary Figure 5. ^{1}H NMR spectrum of Azo-PLLA in $CDCl_3$.

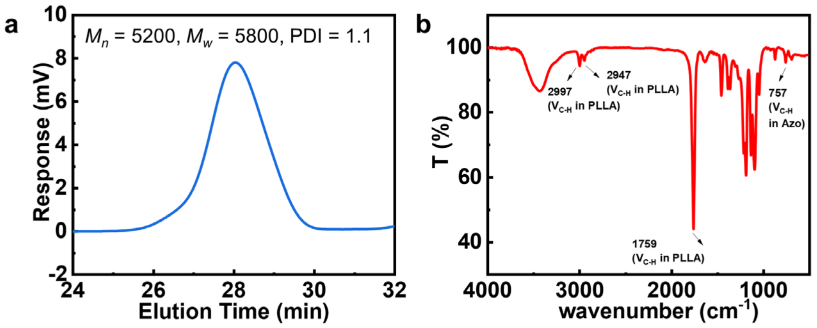

Supplementary Figure 6. (a) SEC spectrum of Azo-PLLA. (b) FTIR spectrum of Azo-PLLA.

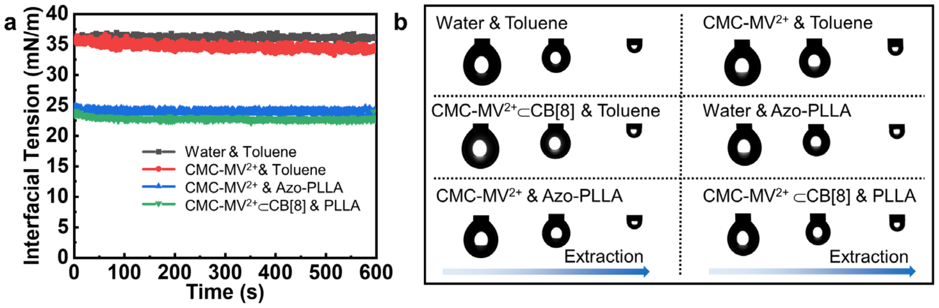

Supplementary Figure 7. (a) Time evolution of interfacial tensions for water & toluene, CMC-MV^{2+} & toluene, CMC-MV^{2+} & Azo-PLLA, and CMC-$MV^{2+}\subset$CB[8] & PLLA. (b) A series snapshots of droplets for different water-oil system under extraction.

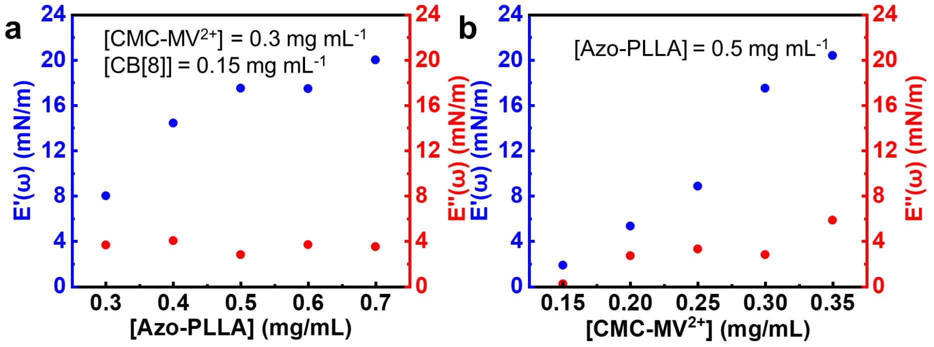

Supplementary Figure 8. Storage and loss dilatational moduli, E'(ω) and E''(ω), of CB[8] surfactant-based assemblies: (a) [CMC-MV^{2+}] = 0.3 mg mL^{-1}, [CB[8]] = 0.15 mg mL^{-1}, [Azo-PLLA] = 0.3-0.7 mg mL^{-1}, ω = 0.2 Hz. (b) [Azo-PLLA] = 0.5 mg mL^{-1}, [CMC-MV^{2+}] = 0.15-0.35 mg mL^{-1}, [CB[8]] = 0.075-0.175 mg mL^{-1}, ω = 0.2 Hz.

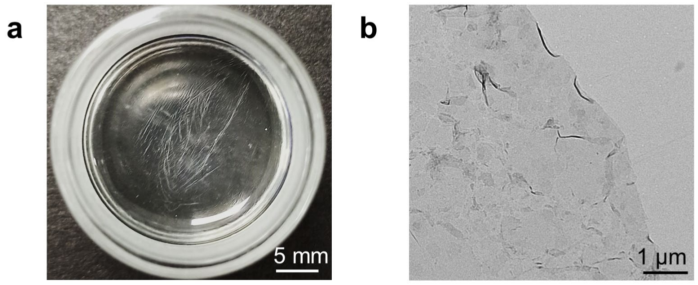

Supplementary Figure 9. (a) Optical image of the 2D films assembled at a flat water-toluene interface. (b) TEM image of the 2D films. [CMC-MV^{2+}] = 0.5 mg mL^{-1}, [CB[8]] = 0.25 mg mL^{-1}, [Azo-PLLA] = 0.5 mg mL^{-1}, assembly time: 20 min.

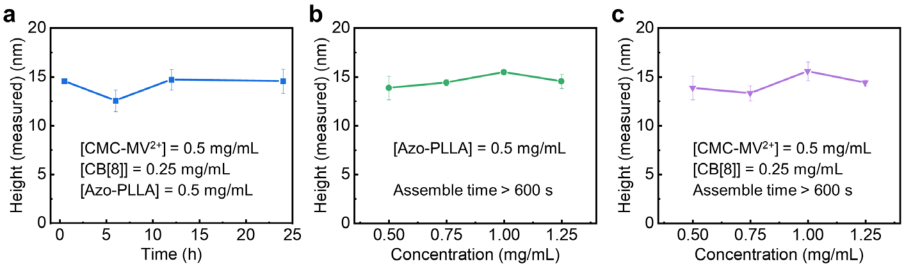

Supplementary Figure 10. (a) Interfacial film thickness as the function of assembled time; [CMC-MV^{2+}] = 0.5 mg mL^{-1}, [CB[8]] = 0.25 mg mL^{-1}, [Azo-PLLA] = 0.5 mg mL^{-1}. (b) Interfacial film thickness as the function of CMC-MV^{2+}/CB[8] concentration; [Azo-PLLA] = 0.5 mg mL^{-1}, [CMC-MV^{2+}] = 0.25-1.25 mg mL^{-1}, [CMC-MV^{2+}] : [CB[8]] = 2:1, Assemble time > 600 s. (c) Interfacial film thickness as the function of Azo-PLLA concentration; [Azo-PLLA] = 0.25-1.25 mg mL^{-1}, [CMC-MV^{2+}] = 0.5 mg mL^{-1}, [CB[8]] = 0.25 mg mL^{-1}, assemble time > 600 s. Values represent the mean, and the error bars represent the SD of the measured values (n = 3).

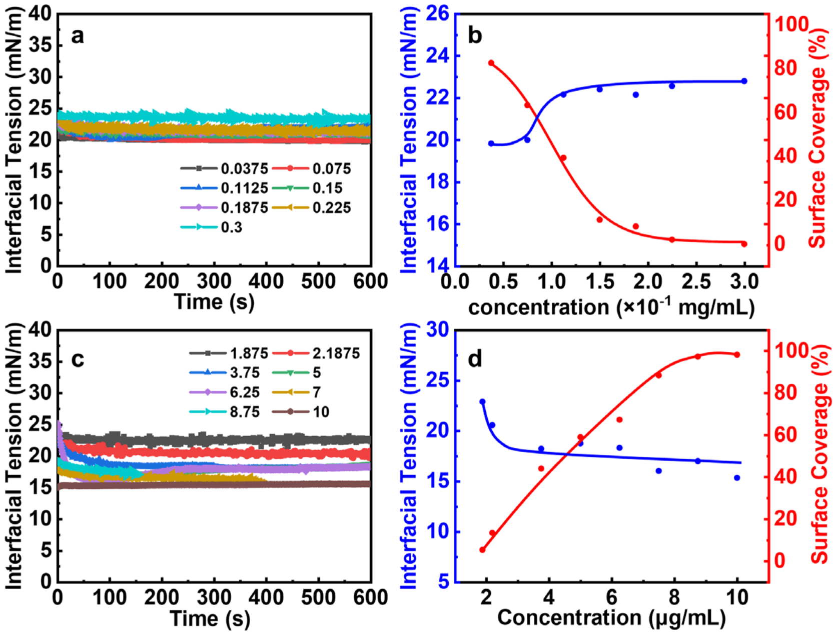

Supplementary Figure 11. (a) Time evolution of interfacial tension by varying the concentration of $Na_2S_2O_4$ in the aqueous phase. (b) Interfacial tension and surface coverage as a function of $Na_2S_2O_4$ concentration. (c) Time evolution of interfacial tension by varying the concentration of NaClO in the aqueous phase. (d) Interfacial tension and surface coverage as a function of NaClO concentration. [CMC-MV^{2+}] = 0.5 mg mL^{-1}, [CB[8]] = 0.25 mg mL^{-1}, [Azo-PLLA] = 0.5 mg mL^{-1}.

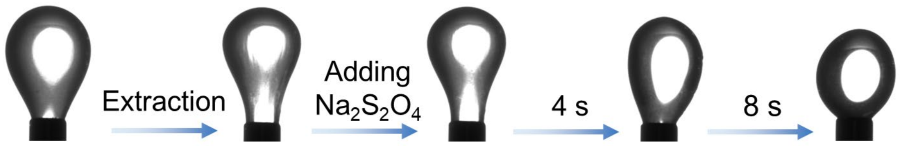

Supplementary Figure 12. Morphology evolution of the pendant droplet with jammed CB[8] surfactants at the interface after inducing $Na_2S_2O_4$ in the aqueous phase.

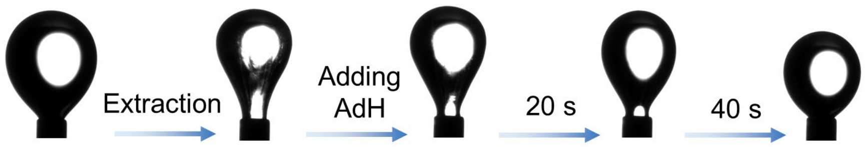

Supplementary Figure 13. Morphology evolution of the pendant droplet with jammed CB[8] surfactants at the interface after inducing AdH in the aqueous phase.

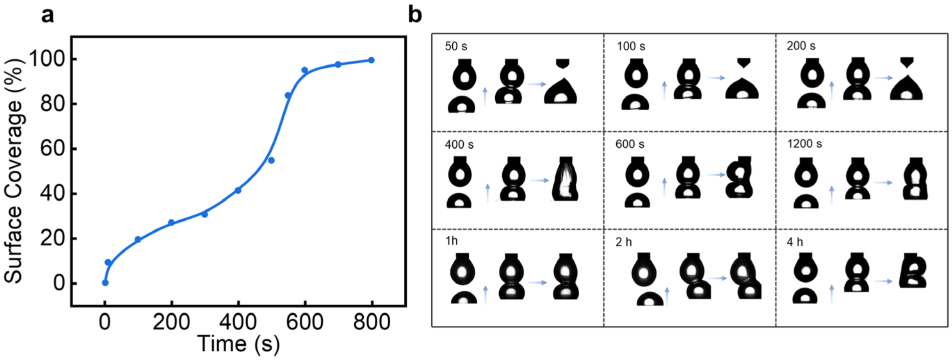

Supplementary Figure 14. (a) Evolution of the surface coverage as a function of time. (b) Optical images showing the process of contact and squeeze of two droplets at different assembly time; [CMC-MV^{2+}] = 0.5 mg mL^{-1}, [CB[8]] = 0.25 mg mL^{-1}, [Azo-PLLA] = 0.5 mg mL^{-1}.

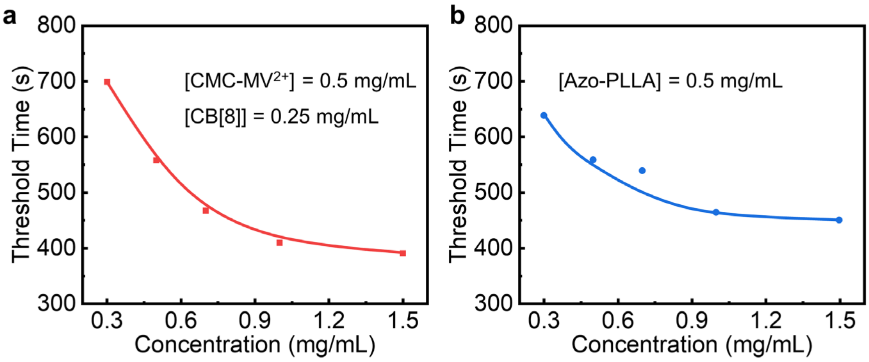

Supplementary Figure 15. (a) Evolution of the threshold time as a function of concentration of Azo-PLLA; [CMC-MV^{2+}] = 0.5 mg mL^{-1}, [CB[8]] = 0.25 mg mL^{-1}. (b) Evolution of the threshold time as a function of concentration of CMC-MV^{2+}/CB[8]; [CMC-MV^{2+}] : [CB[8]] = 2:1, [Azo-PLLA] = 0.5 mg mL^{-1}.

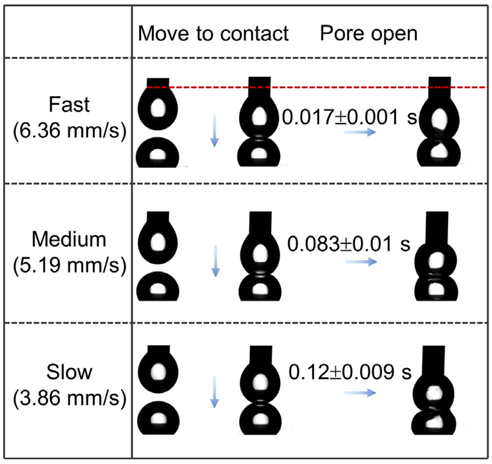

Supplementary Figure 16. Optical images showing the effect of impact velocity on triggering the partial fusion of two droplets. [CMC-MV^{2+}] = 0.5 mg mL^{-1}, [CB[8]] = 0.25 mg mL^{-1}, [Azo-PLLA] = 0.5 mg mL^{-1}.

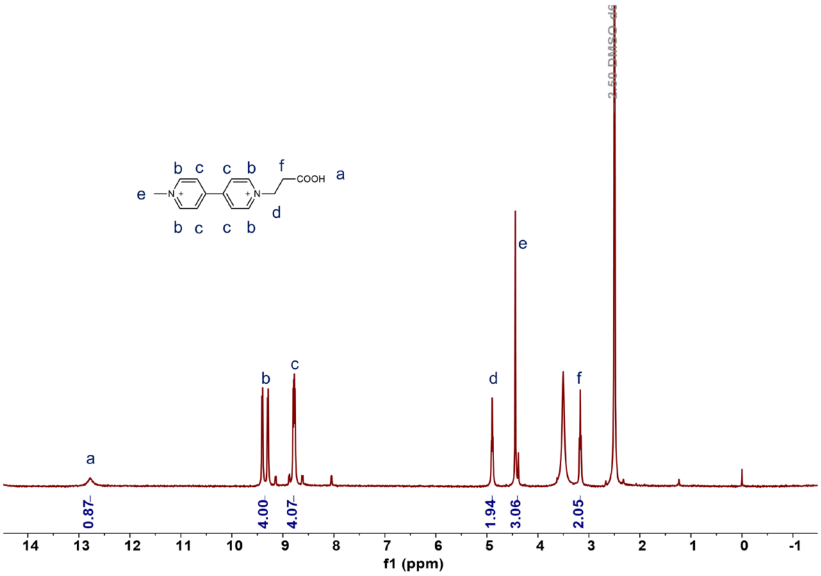

Supplementary Figure 17. ^{1}H NMR spectrum of MV^{2+}-COOH in DMSO-d_6.

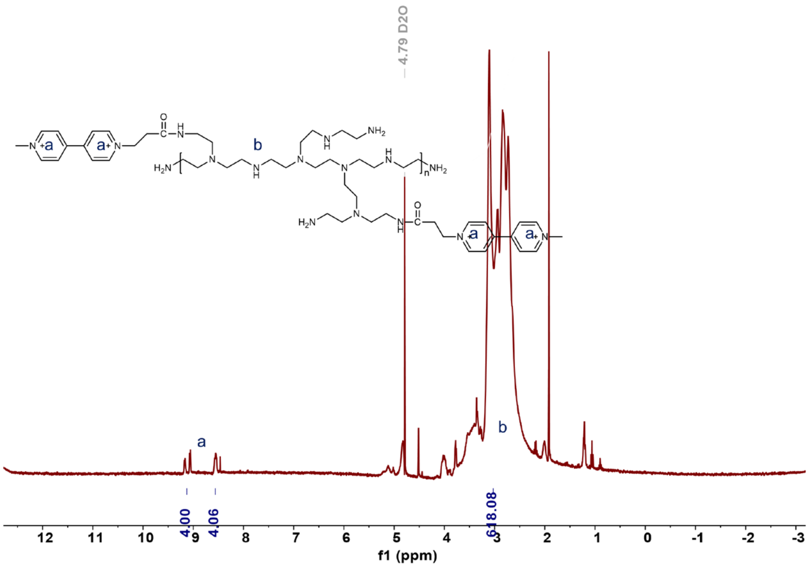

Supplementary Figure 18. ^{1}H NMR spectrum of PEI-MV^{2+} in D_2O.

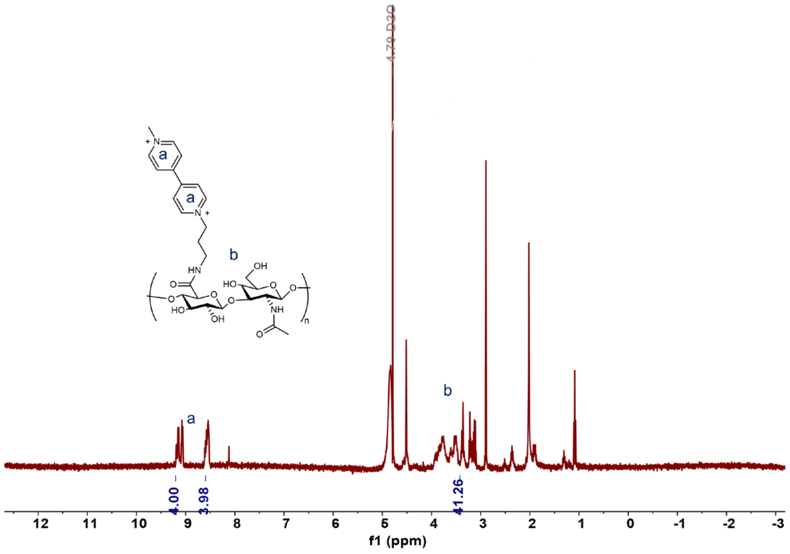

Supplementary Figure 19. ^{1}H NMR spectrum of HA-MV^{2+} in D_2O.

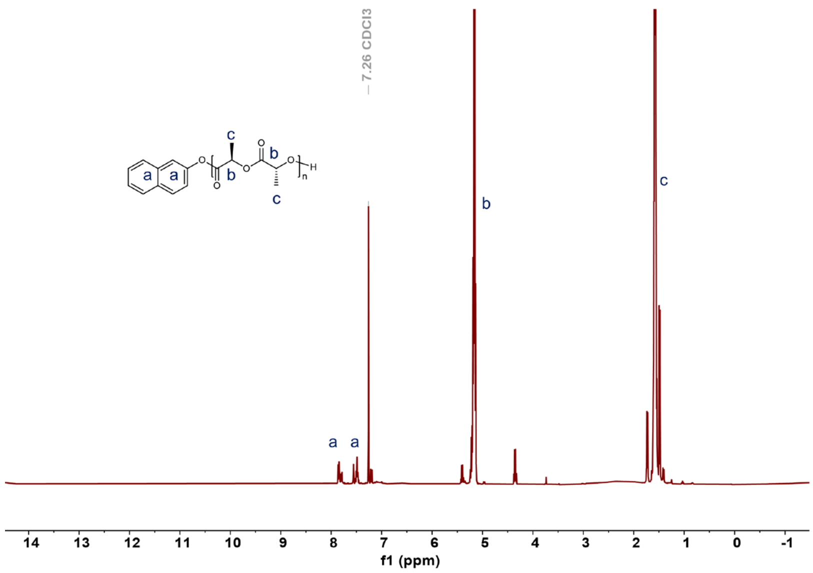

Supplementary Figure 20. ^{1}H NMR spectrum of Nt-PLLA in $CDCl_3$.

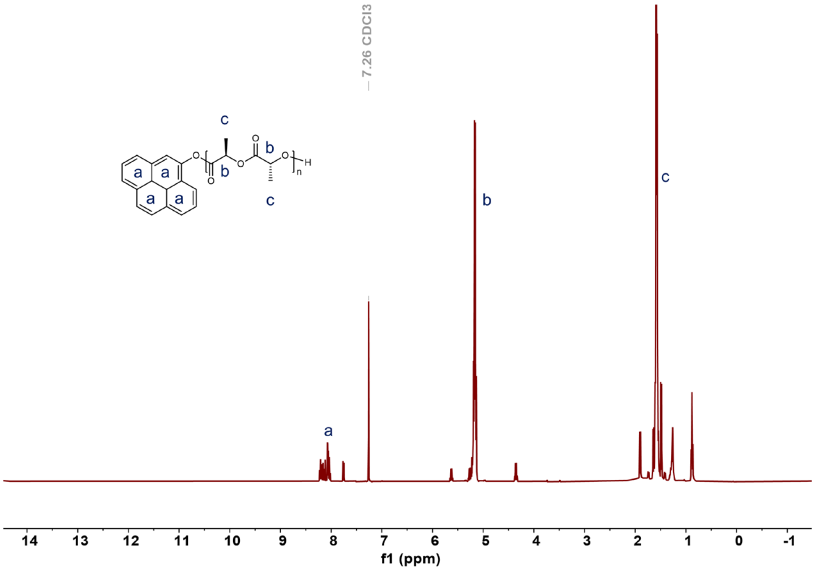

Supplementary Figure 21. ^{1}H NMR spectrum of Py-PLLA in $CDCl_3$.

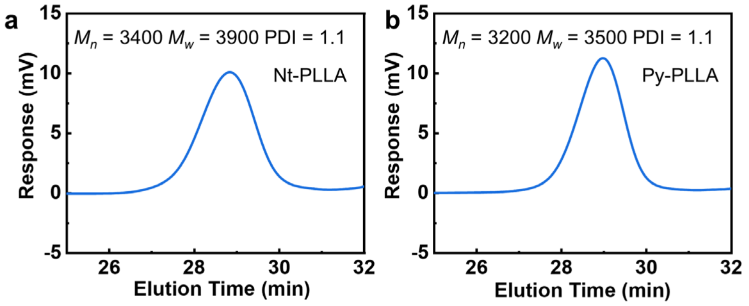

Supplementary Figure 22. SEC spectra of (a) Nt-PLLA and (b) Py-PLLA.

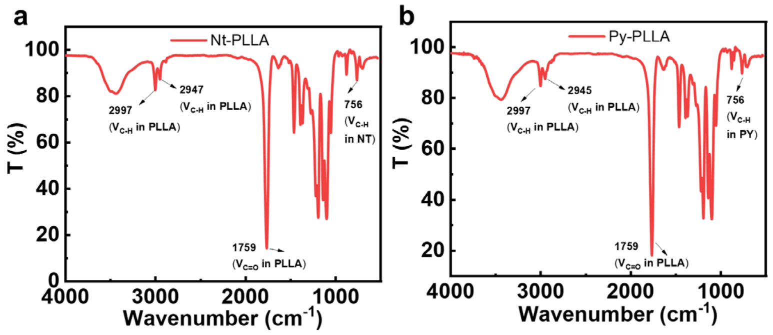

Supplementary Figure 23. FTIR spectra of (a) Nt-PLLA and (b) Py-PLLA.

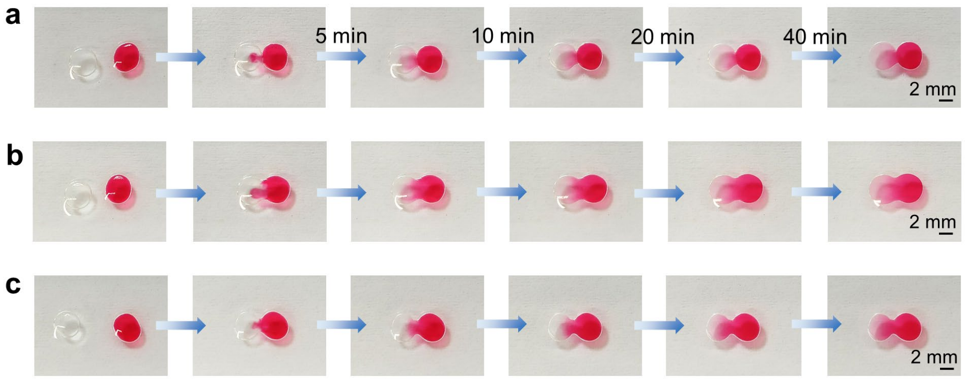

Supplementary Figure 24. Repeatability of the experiment on the diffusion of dye within the droplet networks shown in Fig.4f.

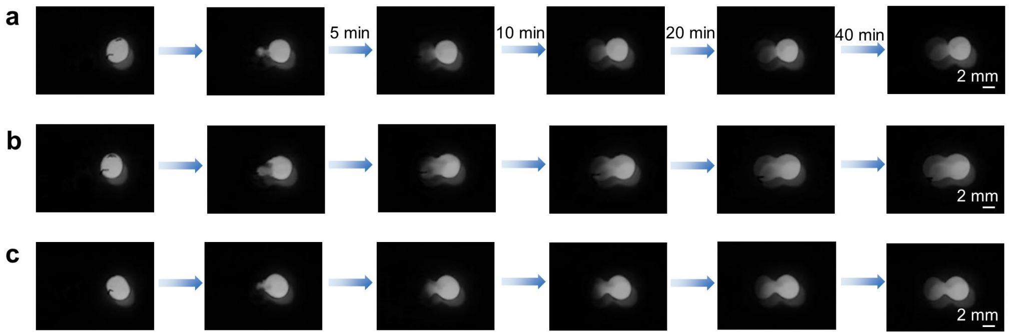

Supplementary Figure 25. Grayscale images of Supplementary Figure 24.

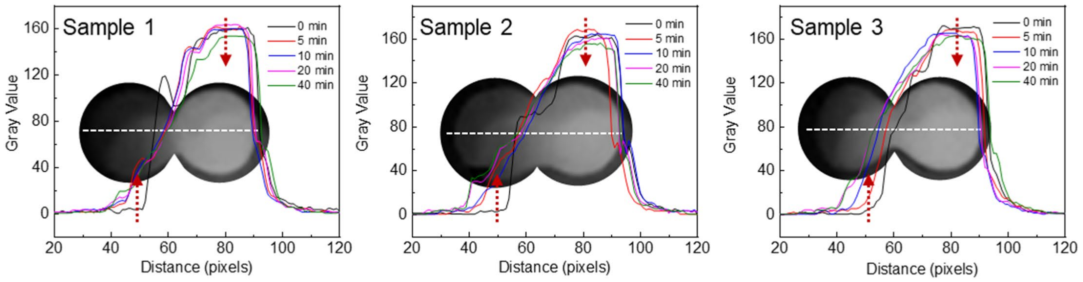

Supplementary Figure 26. Time evolution of the dye diffusion within the droplet networks by plotting the droplet profile (Supplementary Figure 25) with gray values. The gray value is tracked from the middle of the grayscale image.

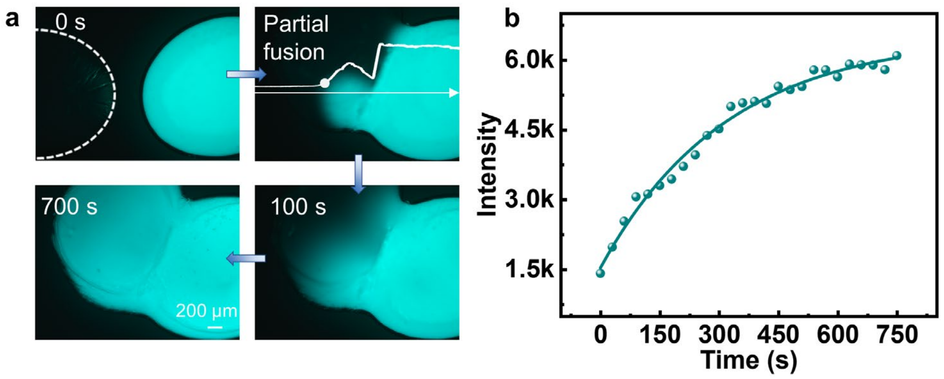

Supplementary Figure 27. (a) Fluorescent images showing the diffusion of dye (Sodium fluorescein) in droplet networks. (b) Time evolution of fluorescence intensity at the white point.

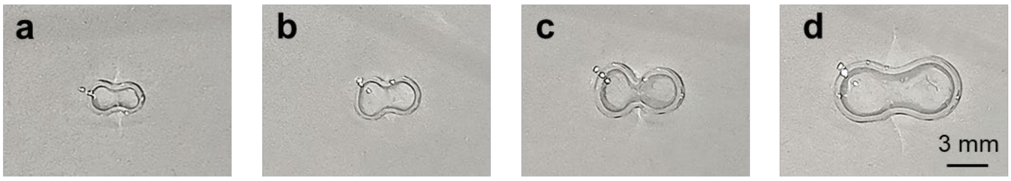

Supplementary Figure 28. Droplet networks constructed by droplet with different volume: (a) 2.5 μL; (b) 5.0 μL; (c) 10.0 μL; (d) 20.0 μL.

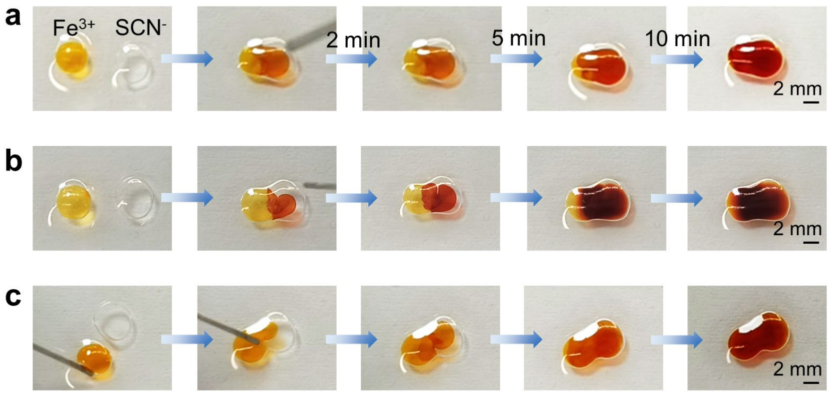

Supplementary Figure 29. Repeatability of experiment on the chromogenic reaction for $Fe^{3+} + 3SCN^{-} = Fe(SCN)_3$ within the droplet networks shown in Fig.5a-b.

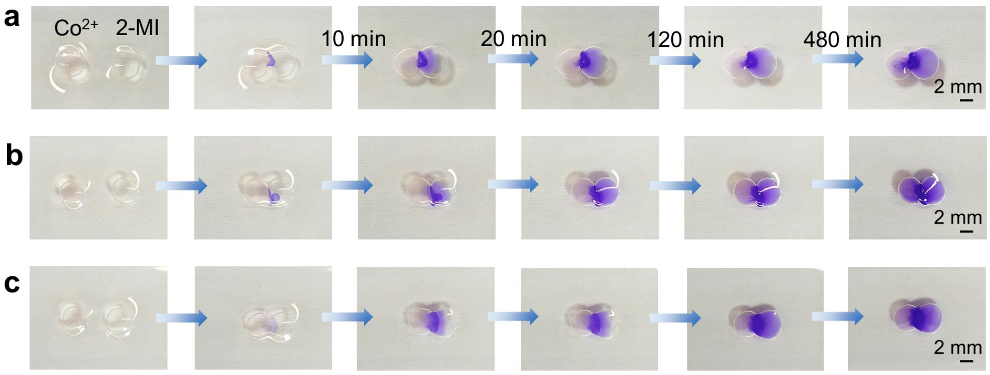

Supplementary Figure 30. Repeatability of the experiment on the synthesis of ZIF-67 within the droplet networks shown in Fig.5c-d.

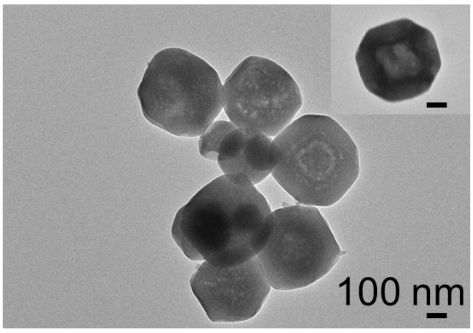

Supplementary Figure 31. The TEM image of ZIF-67.

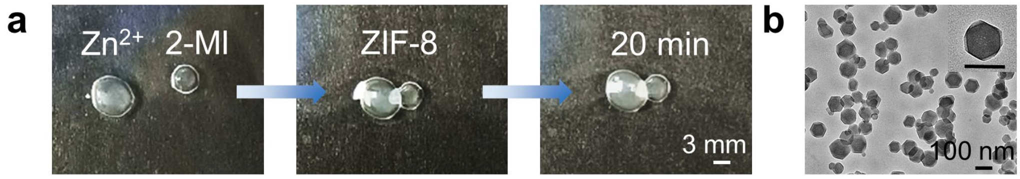

Supplementary Figure 32. (a) Optical images showing the synthesis of ZIF-8 within the snowman like droplet network. (b) The TEM image of ZIF-8.

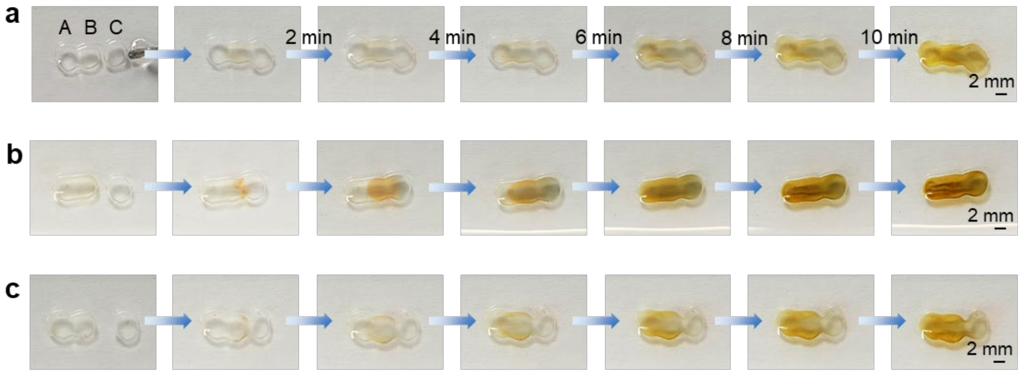

Supplementary Figure 33. Repeatability of the experiments on the cascade enzymatic reaction within the droplet networks shown in Fig.5e-f.

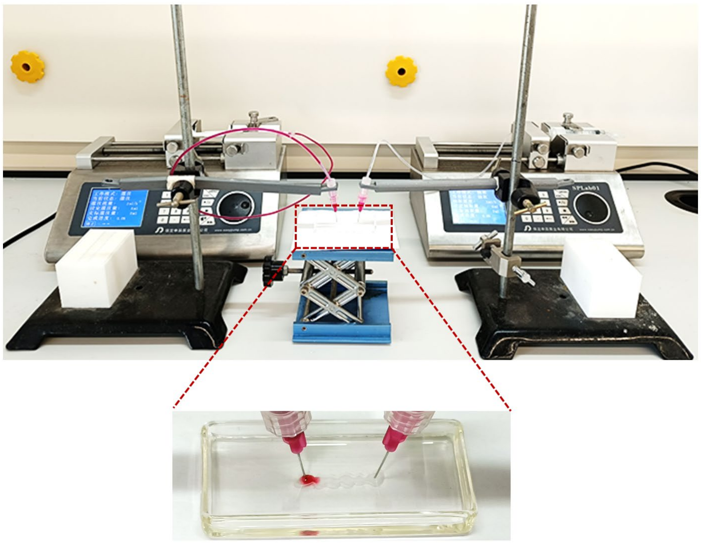

Supplementary Figure 34. Photograph of the continuous flow system based on the droplet network channel.

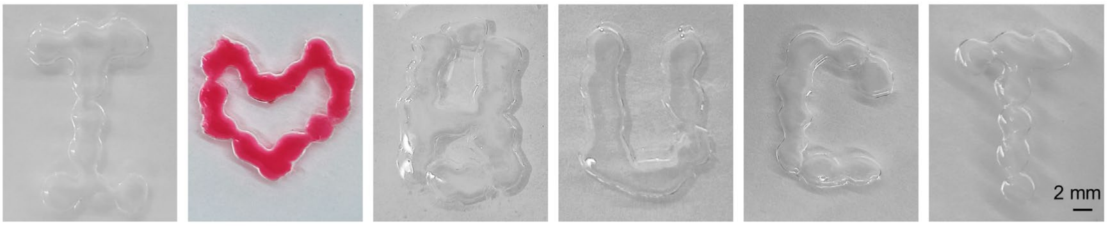

Supplementary Figure 35. Optical images showing different droplet networks including letter I, heart shape, letter B, letter U, letter C, and letter T.

Reference

[1] Rauwald, U. & Scherman, O. A. Supramolecular Block Copolymers with Cucurbit[8]uril in Water. *Angew. Chem. Int. Ed.* **47**, 3950-3953 (2008).

[2] Yin, Z. *et al.* Dissipative Supramolecular Polymerization Powered by Light. *CCS Chem.* **1**, 335-342 (2019).

[3] Sun, S. *et al.* Responsive Interfacial Assemblies Based on Charge-Transfer Interactions. *Angew. Chem. Int. Ed.* **60**, 26363-26367 (2021).

[4] Chen, C.-J., Li, D.-D., Wang, H.-B., Zhao, J. & Ji, J. Fabrication of dual-responsive micelles based on the supramolecular interaction of cucurbit[8]uril. *Polym. Chem.* **4**, 242-245 (2013).

[5] Li, D.-d. *et al.* Cucurbit[8]uril Supramolecular Assembly for Positively Charged Ultrathin Films as Nanocontainers. *Langmuir* **29**, 14101-14107 (2013).
