# Supplementary material for: Reconfigurable droplet networks
Source: Nat Commun. 2024 Feb 5;15:1058. doi: 10.1038/s41467-024-45214-1 (PMC10844234; doi:10.1038/s41467-024-45214-1)
Supplement: Supplementary file 3 — Description of Additional Supplementary Files [file 41467_2024_45214_MOESM3_ESM.docx]

**List and description of supplementary movies**

**Supplementary Movie 1:** The shape evolution of a pendant droplet under a small compression.

**Supplementary Movie 2:** The shape evolution of a pendant droplet in an extraction–reinjection process.

**Supplementary Movie 3:** The diffusion of fluorescent dye within the droplet networks.

**Supplementary Movie 4:** The reconfigurability of the droplet networks.

**Supplementary Movie 5:** The synthesis of Fe(SCN)_3_ within the droplet networks.

**Supplementary Movie 6:** The synthesis of ZIF-67 within the droplet networks.

**Supplementary Movie 7:** The cascade enzymatic reaction within the droplet networks.

**Supplementary Movie 8:** The transmission of a dye solution within the linear droplet network channel.

**Supplementary Movie 9:** The transmission of three different kinds of dye solution within the “Y” type of droplet network channels.
